# Hepatic enhancement at computed tomography: is there a dependence on body weight past institutional contrast dosing limits?

**DOI:** 10.1177/02841851221079014

**Published:** 2022-03-10

**Authors:** Savas Kesen, Anders Svensson, Daniel Thor, Torkel B. Brismar

**Affiliations:** 1 Division of Radiology, Department of Clinical Science, 206106 Intervention and Technology at Karolinska Institutet, Stockholm, Sweden; 2 Department of Radiology, 379654 Södersjukhuset, Stockholm, Sweden; 3 Department of Radiology, Imaging and Physiology, 59562 Karolinska University Hospital, Stockholm, Sweden; 4 Medical Radiation Physics and Nuclear Medicine, Imaging and Physiology, 59562 Karolinska University Hospital, Stockholm, Sweden

**Keywords:** Liver, computed tomography, intravenous contrast agents

## Abstract

**Background:**

Although described in product monographs, the maximum contrast media (CM) dose at computed tomography (CT) varies among institutions.

**Purpose:**

To investigate whether an upper limit of 40 g of iodine in women and 50 g in men is sufficient or if there is a body weight (BW) dependence of mean hepatic enhancement (MHE) beyond those thresholds.

**Material and Methods:**

At our institution, CM injection duration is fixed to 30 s and dosed 600 mg iodine/kg up to 40 g in women and 50 g in men. Pre- and post-contrast hepatic attenuation values (HU) were retrospectively obtained in 200 women and 200 men with glomerular filtration rate >45 mL/min undergoing 18-flurodeoxyglucose PET-CT (18F-FDG PET-CT) of which half weighed below and half above those dose thresholds using iodixanol 320 mg iodine/mL or iomeprol 400 mg iodine/mL. The correlation between BW and MHE was assessed by simple linear regression.

**Results:**

Weight range was 41–120 kg in women and 47–137 kg in men. There was no significant relationship between MHE and BW in women receiving <40 g (r = −0.05, *P* = 0.63) or in men receiving <50 g (r = 0.18, *P* = 0.07). Above those thresholds there was an inverse relationship (r = −0.64, *P*<0.001 in women and r = −0.30, *P*<0.002 in men). There was no apparent upper limit where the dependence of hepatic MHE on BW decreased. Hepatosteatosis limited MHE.

**Conclusion:**

Adjusting CM to BW diminishes the dependence of MHE on BW. There was no apparent upper limit for the relationship between BW and MHE in heavier patients at CM-enhanced CT.

## Introduction

Contrast media (CM)-enhanced computed tomography (CT) has become integral in diagnosing hepatic pathology such as hepatocellular carcinoma (HCC) and hepatic metastases (1). At a minimum, a relative mean hepatic enhancement (MHE) of 50 Hounsfield units (HU) after administration of CM has been recommended in order to facilitate correct diagnostics (2). Several CM-related factors such as CM dose and injection speed and patient-related factors such as patient habitus and cardiac function affect hepatic CM enhancement (2–6). Among the patient-related factors, patient habitus, i.e. extracellular volume, has been described as the most important for hepatic enhancement (7).

Several studies have shown an inverse correlation between patient size indices as surrogates for extracellular volume, such as body weight (BW), lean body mass (LBM), body surface area (BSA), and hepatic enhancement (8–21). This has led to the employment of weight-based protocols. An upper limit is often used in these protocols due to the reasoning that extra weight beyond the limit is caused by adipose tissue. According to that reasoning, any additional CM dose would not increase the diagnostic sensitivity but increase the risk of post-contrast acute kidney injury (PC-AKI). Although the risk of PC-AKI has been stated to be dose related (22), the causality between CM and PC-AKI is still debated with growing uncertainty around the existence or relevance of PC-AKI (23–25). Furthermore, there is insufficient evidence that PC-AKI is dose related when administering CM intravenously compared to intra-arterially (26).

When using a weight-based dosing protocol, an upper limit is often applied, either strictly weight based, e.g. 80 kg in women and 100 kg in men, or a certain amount of iodine, e.g. 40 to 50 g. There are several reasons for using an upper limit. One is practical – there is a physical limit on how fast the power injector can deliver the CM without risking extravasation. Typically, CM is not injected faster than 5 mL/s through a 20-G cannula, resulting in a practical limit of 60 g of iodine when injecting 400 mg I/mL during 30 s. However, when necessary, CM can be injected through both arms or through cannulas of greater size. Second, as mentioned previously, there is also a risk of PC-AKI to consider. Third, it can be reasoned that additional weight past these limits does not increase blood or extracellular volume as it most often is due to excess adipose tissue, which is poorly vascularized. However, to our knowledge, there are no published data that any such thresholds really exist. Consequently, the use of upper limits should be questioned because their use poses concern for non-diagnostic hepatic enhancement. Thus, the aim of the present study was to investigate whether there is such an upper threshold or if there is a body weight (BW) dependence of mean MHE beyond currently applied contrast medium dose limits.

## Material and Methods

**Fig. 1.**
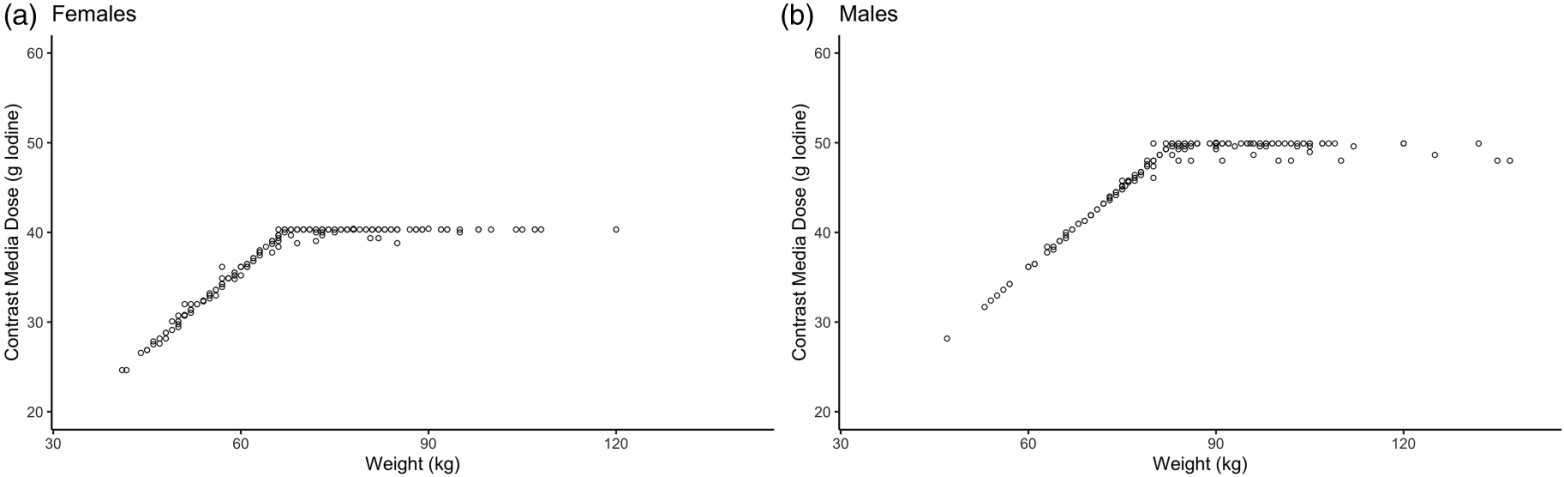
The CM dosing strategy in (a) women and (b) men. The CM dose was increased by 600 mg iodine per kg up to a limit of 40 g iodine in women (i.e. until BW 66.7 kg) and to 50 g iodine in men (i.e. until BW 83.3 kg). Each dot represents the actual given individual dose. BW, body weight; CM, contrast media.

After institutional and ethical approval (Swedish Ethical Review Authority, reference no. 2019-03495), with a waiver of informed consent, patients who had undergone 18-flurodeoxyglucose PET-CT (18F-FDG PET-CT) between 30 November 2018 and 1 June 2017 at the present institution were identified. This patient cohort was deemed suitable as they, as a part of the PET examination, undergo a low-radiation dose non-contrast-enhanced CT. The so-called attenuation correction CT (ACCT) is not used for diagnostic purposes, but for optimizing the PET signal. In the present study, the ACCT was used to evaluate native hepatic attenuation. At our institution, all patients undergoing a PET-CT undergo a full, diagnostic thoraco-abdominal CT in the parenchymal phase. According to the protocol, the CM dose is compensated for BW with 600 mg iodine per kg up to a limit of 40 g iodine in women and 50 g iodine in men, provided that their glomerular filtration rate (GFR) is >45 mL/min (Fig. 1). This corresponds to 66.7 kg in women and 83.3 kg in men. There is no compensation for any additional BW. Thus, the patients can be said to be assigned to four different groups consisting of women or men above or below these thresholds. To retrospectively identify patients above the thresholds, denoted fixed-dose groups, the medical records of the first 100 women receiving 40 ± 2 g and first 100 men receiving 50 ± 2 g from 30 November 2018 and backwards were retrospectively reviewed. For comparison, 100 women and 100 men weighing less, receiving CM dosage based on BW, were selected by identifying the first 10 cases in each weight decile. Those were denoted weight-adjusted groups. Patients who could not follow the CM protocol due to insufficient renal function were replaced until 100 patients were identified in each group. When collecting data, 7 women and 46 men in the fixed-dose groups had to be replaced, and in the weight-adjusted groups 9 women and 28 men were replaced (Table 1). Age, BW, amount of CM, and study date were recorded.

**Table 1.** The number of patients screened, number excluded due to not receiving CM according to protocol with GFR <45 mL/min, and final included number of patients.

	Women		Men	
	Weight-adjusted	Fixed-dose	Weight-adjusted	Fixed-dose
Screened	109	107	128	146
Excluded	9	7	28	46
Included	100	100	100	100

Values are given as n.

CM, contrast media; GFR, glomerular filtration rate.

All images were acquired on a PET-CT imaging system (Siemens Biograph128-mCT; Siemens Healthineers, Erlangen, Germany). Before CM injection, a low radiation dose scan was obtained using a quality reference mAs of 10 mAs at 120 kV. For CM-enhanced imaging, iodixanol 320 mg iodine/mL (Visipaque ®-320; GE Healthcare, Chalfont St Giles, UK) or iomeprol 400 mg iodine/mL (Iomeron®-400; Bracco Imaging SpA, Milan, Italy) was delivered through a 20-G cannula placed in an antecubital vein and delivered with a fixed duration of 30 s using a power injector (Stellant Dual Head Injector; Medrad, Bayer, Pittsburgh, PA, USA) followed by a saline flush of 50 mL. The portovenous phase was started 45 s after a threshold of 160 HU was obtained in a region of interest (ROI) placed in the aorta at the level of the diaphragm. Imaging was obtained at 120 kV and quality reference mAs of 180. Pre- and post-contrast images were reconstructed to a slice thickness of 5 mm.

Attenuation measurements (in HU) of the hepatic parenchyma before and after intravenous CM were quantified using a dedicated workstation (Sectra PACS; Sectra AB, Linköping, Sweden) by placing a circular ROI at least 10 mm in diameter at three different levels; liver hilum, on an image slice 30 mm caudally to the hemidiaphragm; and on an image slice 30 mm cranially to the caudal edge of the liver. The obtained values were averaged. ROIs were placed centrally, typically in segments 4, 5, and 8 by a radiology resident with one year of experience (SK). Care was taken to avoid major vessels and pathological tissue and to evaluate the same volume before and after contrast. The number of patients with hepatosteatosis, defined as <40 HU before contrast (27) was noted. The MHE was calculated by subtracting pre-contrast hepatic attenuation from post-contrast hepatic attenuation, i.e. *MHE* *=* *mean of post-contrast ROIs – mean of pre-contrast ROIs*.

Means with standard deviation and range were used for descriptive data such as age, BW, CM dose, and MHE. The two-sample *t*-test for independent samples was used to compare means. Correlations between the BW and measurement values were analyzed with Pearson's correlation coefficient (r) using a simple linear regression model. Hepatic enhancement of <30 HU was deemed non-diagnostic, whereas 30–49 HU was deemed suboptimal, and ≥50 HU was deemed optimal. Fisher's exact test was used to compare the number of optimal, suboptimal, and non-diagnostic examinations in addition to number of cases with hepatosteatosis and those without. All statistical analyses were conducted in R Version 3.5.3 (R Foundation for Statistical Computing, Vienna, Austria). *P* < 0.05 was considered significant.

## Results

**Fig. 2.**
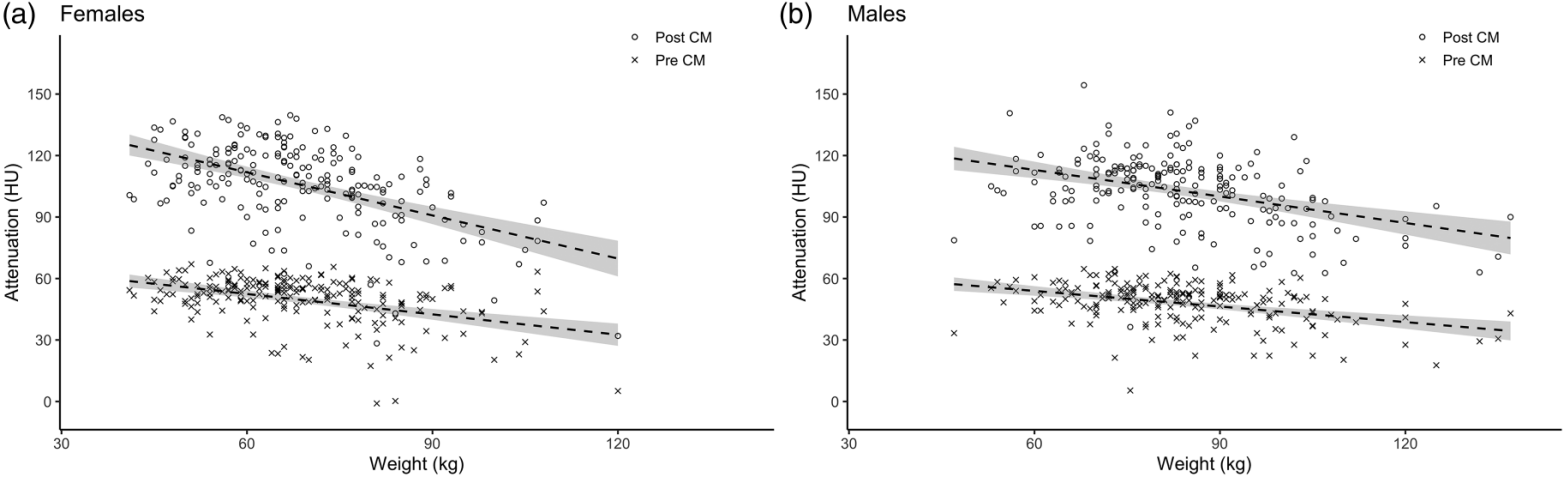
(a, b) Hepatic attenuation plotted as a function of BW before and after CM with separately fitted linear regression lines with 95% confidence intervals for women and men, respectively. BW, body weight; CM, contrast media.

In total, 400 patients (age range = 19–91 years; weight range = 41–137 kg) were analyzed. The attenuation of hepatic parenchyma was significantly lower (Table 2) in the fixed-dose groups compared to the weight-adjusted ones, both before and after contrast (Figure 2).

**Table 2.** Patient groups with sex, age, body weight, hepatic attenuation, MHE, and number of patients with hepatosteatosis.

	Women			Men		
	Weight-adjusted	Fixed-dose	*P*	Weight-adjusted	Fixed-dose	*P*
N	100	100		100	100	
Age (years)	63 ± 15 (19–91)	63 ± 13 (26–88)	0.90	63 ± 15 (22–88)	63 ± 11 (20–81)	0.99
Weight (kg)	57 ± 6.7 (41–66)	80 ± 11 (67–120)	<0.001	72 ± 8.0 (47–82)	95 ± 12 (83–137)	<0.001
Total dose (g)	34 ± 4.0 (25–40)	40 ± 0.3 (39–40)	<0.001	43 ± 4.7 (28–50)	50 ± 0.6 (48–50)	<0.001
Hepatic attenuation before contrast (HU)	53 ± 8.2 (23–67)	46 ± 13 (−1.0–66)	<0.001	51 ± 8.8 (5.4–65)	45 ± 10 (18–62)	<0.001
Hepatic attenuation after contrast (HU)	113 ± 16 (62–139)	99 ± 21 (28–140)	<0.001	108 ± 16 (36–154)	98 ± 17 (63–137)	<0.001
MHE (HU)	59 ± 12 (28–86)	53 ± 12 (25–84)	<0.001	57 ± 11 (31–108)	53 ± 12 (19–86)	0.005
Hepatosteatosis (%)	6 (6)	24 (24)	<0.001	9 (9)	25 (25)	0.005
Non-diagnostic (%)	1 (1)	5 (5)	0.21	0 (0)	2 (2)	0.50
Suboptimal (%)	26 (26)	33 (33)	0.35	22 (22)	44 (44)	0.002
Optimal (%)	73 (73)	62 (62)	0.13	78 (78)	54 (54)	<0.001

Values are given as n (%) or mean ± SD (range). One standard deviation and range have been written out. *P* values refer to the difference between weight-adjusted and fixed-dose groups for women and men, respectively.

HU, Hounsfield units; MHE, mean hepatic enhancement.

### Women

**Fig. 3.**
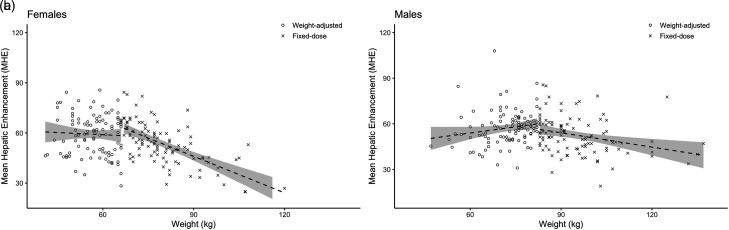
(a, b) MHE plotted as a function of BW for the weight-adjusted and fixed-dose groups for women and men, respectively. Linear regression lines with 95% confidence intervals have been fitted for each group separately. BW, body weight; MHE, mean hepatic enhancement.

MHE was >50 HU in both the fixed-dose and the weight-adjusted female groups, but the enhancement was weaker in the fixed-dose group (53 ± 12 HU) than in the weight-adjusted group (59 ± 12 HU, *P* < 0.001). There was a significant relationship between BW and MHE in the fixed-dose group (r = −0.64, *P* < 0.001) but not in the weight-adjusted group (r = −0.05, *P* = 0.63) (Fig. 3a). In the fixed-dose group, 38 patients had MHE <50 (five non-diagnostic, 33 suboptimal), while the number was 27 (one non-diagnostic, 26 suboptimal) in the weight-adjusted group, but the difference was not statistically significant (*P* = 0.13).

### Men

Mean hepatic enhancement >50 HU was achieved in both fixed-dose and weight-adjusted male groups, but the enhancement was weaker in the fixed-dose group (53 ± 12 HU and 57 ± 11 HU, respectively; *P* = 0.005). MHE correlated significantly with BW (r = −0.30, *P* = 0.002) in the fixed-dose group (Fig. 3b). No statistically significant relationship was seen between BW and contrast enhancement in the weight-adjusted group (r = 0.18, *P* = 0.07). There were more patients with MHE <50 HU in the fixed-dose group (two non-diagnostic, 44 suboptimal) than in the weight-adjusted group (0 non-diagnostic, 22 suboptimal; *P* < 0.001).

## Discussion

In the present study, there was a considerable relationship between BW and hepatic enhancement in women when CM dose was not compensated for BW >66.7 kg. A similar but less prominent relationship was observed in men weighing >83.3 kg. Thus, when aiming for a hepatic enhancement >50 HU there seems to be no apparent upper weight threshold when CM dose should no longer be compensated.

When CM dose was compensated for BW, i.e. in patients weighing <66.7 kg and 83.3 kg respectively, there was no statistically significant relationship between BW and MHE for women and men alike, further supporting the idea that adjusting iodine dose to BW leads to a more consistent enhancement of hepatic parenchyma (20,21). However, in the male weight-adjusted group there was a trend of increasing MHE with increasing BW suggesting overcompensation (Fig. 3b). This might suggest that the relationship between BW and MHE is not linear in men. To better dose CM in men, the compensation of iodine dose for BW should probably be reduced with increasing weight or a fixed baseline dose to which a weight-based compensation could be added. On the other hand, the risk of errors in clinical practice might increase when using more complex CM dosing algorithms.

Based on the findings of this study, there is no apparent upper limit on CM dosage. However, from a nephrological perspective it has been recommended that the CM dose should be kept within safe limits to decrease the risk of PC-AKI (28). According to those recommendations, the total amount of iodine should not exceed the GFR with a factor of 1, or 0.5 when <45 mL/min. That GFR limit will probably affect a rather large proportion of heavier men, as 46 (31%) of those screened for this retrospective study had to be excluded due to CM dose restrictions. In women, this upper limit is less prominent as they weigh less. In this study only 7 (7%) had to be excluded for that reason. Pharmaceutical companies also have maximum dosages listed on their product monographs, so exceeding these dosages would be off-label. Higher doses require faster injection rates when delivered at a fixed injection duration, and higher injection rates require larger bore cannulas and are associated with increased interstitial injections. It may also be discussed that higher CM doses would increase the cost of the CM. However, compared to the cost of recalling patients due to non-diagnostic exams or, worse, accepting a suboptimal scan, the cost of the CM should not be an issue.

Mean hepatic enhancement was >50 HU in all four groups, although this unfortunately does not translate to all patients achieving optimal diagnostic hepatic enhancement. This is due to the relatively high variation in enhancement among patients, resulting in 9 (2%) patients having non-diagnostic examinations and 135 (34%) having suboptimal examinations. Besides differences in body composition and cardiac output, cirrhosis affects hepatic attenuation and enhancement (7). As shown in Fig. 2, there was a significant inverse relationship between BW and hepatic attenuation before contrast, which would be consistent with higher fat content at a higher weight. To our knowledge, it has not previously been shown that steatosis limits hepatic parenchymal enhancement. A probable explanation for less hepatic enhancement might be that there is less extracellular volume at hepatosteatosis and cirrhosis. Similarly, the fibrous transformation of the liver in cirrhosis influences the portal circulation affecting the distribution of CM and thereby enhancement.

The present study has some limitations. First, the study was retrospective, with the inherent risk of selection bias and uncertainty on recorded parameters. Second, patients with cirrhosis or hepatosteatosis were not excluded, leading to lower mean hepatic enhancement and weaker observed relationships. However, the inclusion of these patients meant that the study cohort more closely mirrored the patient population PET-CT/CT is intended for. The influence from fibrosis should also be low in our study as PET-CT is not a part of primary liver cancer evaluation and the incidence in the studied population is low, about 14/100,000 (29). Our data were collected on a patient group undergoing PET-CT, which has a greater prevalence of cancer and chemotherapy compared to patients undergoing routine abdominal CT. Third, data on cardiac output were not collected. However, the confounding effect of cardiac output should be negligible due to the use of bolus tracking during image acquisition. Patients with impaired renal function, in total 90 (18%) of the 490 screened, were excluded from the study due to not receiving iodine according to the dosing algorithm. Thus, the results might not be applicable to patients with impaired renal function.

In conclusion, there appears to be no upper threshold where hepatic enhancement ceases to be dependent on increasing BW. After consideration of kidney function, an iodine dose adjusted to BW, or a surrogate, without an upper limit may be considered in order to achieve a more consistent hepatic enhancement and to reduce the risk of suboptimal scans.
